# Anacardic Acid Enhances the Proliferation of Human Ovarian Cancer Cells

**DOI:** 10.1371/journal.pone.0099361

**Published:** 2014-06-12

**Authors:** Yin-Ling Xiu, Yang Zhao, Wen-Feng Gou, Shuo Chen, Yasuo Takano, Hua-Chuan Zheng

**Affiliations:** 1 Department of Gynecology, The First Affiliated Hospital of China Medical University, Shenyang, P.R. China; 2 Department of Biochemistry and Molecular Biology, Institute of Pathology and Pathophysiology, College of Basic Medicine, China Medical University, Shenyang, P.R. China; 3 Clinical Cancer Institute, Kanagawa Cancer Center, Yokohama, Japan; University of Navarra, Spain

## Abstract

**Background:**

Anacardic acid (AA) is a mixture of 2-hydroxy-6-alkylbenzoic acid homologs. Certain antitumor activities of AA have been reported in a variety of cancers. However, the function of AA in ovarian cancer, to date, has remained unknown.

**Methods:**

Ovarian cancer cell lines were exposed to AA, after which cell proliferation, apoptosis, invasion and migration assays were performed. Phalloidin staining was used to observe lamellipodia formation. Reverse transcription polymerase chain reaction (RT-PCR) and western blotting were used to assess the mRNA and protein expression levels of Phosphatidylinositol 3-kinase (PI3K), vascular endothelial growth factor (VEGF) and caspase 3.

**Results:**

Our results showed that AA promotes ovarian cancer cell proliferation, inhibits late apoptosis, and induces cell migration and invasion, as well as lamellipodia formation. AA exposure significantly up-regulated PI3K and VEGF mRNA and protein expression, while, in contrast, it down-regulated caspase 3 mRNA and protein expression in comparison to untreated control cells.

**Conclusion:**

Taken together, our results demonstrate for the first time that AA may potentiate the proliferation, invasion, metastasis and lamellipodia formation in ovarian cancer cell lines via PI3K, VEGF and caspase 3 pathways.

## Introduction

Ovarian cancer remains the most common cause of death from gynecological malignancies [1], [2]. Primary treatment of ovarian cancer is surgical resection of visible disease followed by adjuvant chemotherapy, usually consisting of a combination of platinum-based and taxane-based chemotherapy. Given that recurrence and metastasis seriously affect the prognosis of ovarian cancer, the five-year survival rate for all stages of ovarian cancer has been estimated to be 35–38% [3], [4]. Thus, the study of new second-line chemotherapy drugs, which inhibit the metastatic and recurrence processes of ovarian cancer, have become the focus of recent research interest as their development may improve five-year survival rates.

Anacardic acid (AA) is a mixture of 2-hydroxy-6-alkylbenzoic acid homologs and is commonly found in plants of the Anacardiaceae family [5], [6]. It is a dietary component found in cashew apple (*Anacardium occidentale*) and ginkgo (*Ginkgo biloba*) leaves and fruits. AA acts as a mitochondrial decoupler of oxidative phosphorylation [7] and sensitizes human tumor cells to ionizing radiation by inhibition of histone acetyltransferase activity [8]. AA has also shown certain antitumor activities in prostate cancer, lung carcinoma and breast carcinoma, as well as some other cancers, and is thought to exert its action via various mechanisms [9]–[13]. Recently, an increasing number of studies have investigated the role of AA in cancer, with the hope that it can be eventually applied in clinical treatment. However, the function of AA in ovarian cancer has remained unknown. Thus, our study demonstrates the role and potential mechanisms of AA in ovarian cancer for the first time.

## Materials and Methods

### Cell culture

Ovarian carcinoma cell lines, SKOV3 (serous papillary cystic adenocarcinoma), HO8910 (serous cystic adenocarcinoma) and highly invasive HO8910 (HO8910-PM), were purchased from ATCC. Cisplatin-resistant SKOV3 (SKOV3/DDP) was purchased from the Tumor Cell Bank of the Chinese Academy of Medical Science (Peking, China). Cells were maintained in RPMI 1640 medium (HO8910, HO8910-PM and SKOV3/DDP) and McCoy's 5A medium (SKOV3) supplemented with 10% fetal bovine serum (FBS), 100 units mL^−1^ penicillin (SKOV3/DDP was also supplemented with 20 ng mL^−1^ Cisplatin, (DDP) and 100 µg mL^−1^ streptomycin. Cell lines were kept in a humidified atmosphere of 5% CO_2_ at 37°C with or without AA treatment (Sigma, Saint Louis, America). All cells were harvested by centrifugation after cells were exposed to trypsin, rinsed with phosphate buffered saline (PBS) and subjected to total protein extraction by sonication in radio-immunoprecipitation assay (RIPA) buffer.

### Proliferation assay

Cell Counting Kit-8 (CCK-8, Xiongben, Japan) was employed to determine the number of viable cells via a colorimetric assay. Briefly, 2.5×10^3^ cells/well were seeded to a 96-well plate and allowed to adhere. At different time points, 10 µL of CCK-8 solution was added into each well of the plate and the plate was subsequently incubated for 3 h at 37°C prior to recording of the optical density at 450 nm.

### Cell cycle analysis

After incubation for 48 h at 37°C in an atmosphere of 5% CO_2_, cells were detached by trypsinization, collected, washed twice with PBS and fixed in 10 mL ice-cold ethanol (70%) for at least 2 h. The cells were washed twice with PBS again and incubated with 500 µL RNase (0.25 mg mL^−1^) at 37°C for 30 min. The cells were pelleted, resuspended in propidium iodide (PI) at a concentration of 50 µg mL^−1^ and incubated in the dark at 4°C for 30 min. Cell cycle analysis was performed by analysis of PI staining by flow cytometry.

### Apoptosis assay

Flow cytometry was performed following staining with PI and FITC-labeled annexin V (KeyGEN Biotech, Nanjing, China) according to the manufacturer's protocol to detect phosphatidylserine externalization as an endpoint indicator of early apoptosis in the cells. Briefly, after incubation for 48 h at 37°C in an atmosphere of 5% CO_2_, cells were washed twice with ice-cold PBS, resuspended in 1× binding buffer at a concentration of 1×10^6^ cells mL^−1^ and then incubated with 200 µL 1× binding buffer and 10 µL FITC-annexin V. Samples were gently vortexed and incubated for 15 min at 25°C in the dark, then 300 µL 1× binding buffer and 5 µL PI were added to each tube. Samples were gently vortexed and incubated for less than 1 h at 25°C in the dark. Flow cytometry was performed within 1 h of incubation.

### Wound-healing assay

Cells were seeded at a density of 1.0×10^6^ cells/well in 6-well culture plates. After they had grown to confluency, the cell monolayer was scratched with a pipette tip (200 µL) to create a scratch. The cells were then washed with PBS three times and cultured in FBS-free medium. Cells were photographed at 0, 12, 24, 48 and 72 h (*n* = 9) and the scratched areas were measured using Image software. The wound-healing rate was calculated according to the following formula:

Wound-healing rate  =  (Area of original wound – Area of actual wound at different times)/Area of original wound ×100%.

### Cell invasion assays

For the invasion assay, 5×10^4^ cells were resuspended in FBS-free RPMI-1640 and seeded into the top chambers of Matrigel-coated Transwell inserts (BD Bioscience, San Jose, CA, USA). The lower compartment of the chamber contained 10% *v/v* FBS as a chemoattractant. After incubation for 48 h at 37°C in an atmosphere of 5% CO_2_, the cells on the upper surface of the membrane were wiped away, and the cells on the lower surface of the membrane were washed with PBS, fixed in 100% methanol and stained with crystal violet dye to quantify the extent of invasion.

### Real-time reverse transcription polymerase chain reaction (real-time RT-PCR)

Total RNA was extracted from the ovarian carcinoma cell lines using TRIzol (Takara, Kyoto, Japan). Real-time RT-PCR was performed from 2 µg of total RNA using AMV reverse transcriptase and random primers (Takara, Kyoto, Japan). PCR primers were designed according to the sequences in GeneBank and are listed in Table S1. Amplification of cDNA was performed according to the manufacturer's protocol using an SYBR Premix Ex Taq II kit (Takara, Kyoto, Japan) and *GAPDH* as an internal control. Briefly, RT-PCR amplification of cDNA for each primer was carried out in a final volume of 20 µL, containing 10 µL SYBR Premix Ex Taq (×2), 0.08 µL primers, 0.4 µL ROX reference dye and 1 µL template cDNA (50 µg µL^−1^). The protocol parameters were as follows: initial incubation at 95°C for 30 s followed by 40 cycles of denaturation at 95°C for 5 s and annealing at 60°C for 34 s. All the PCR experiments were accompanied with a no-template control and *GAPDH* as an internal control. The relative gene expression level (the amount of target normalized to the endogenous control gene) was calculated using the comparative CT method: 2^−ΔΔCt^. The sequences of primers for real-time quantitative PCR are supplied in Table S1.

### Western blot analysis

Protein assays were performed according to the Bradford method using the Bio-Rad protein assay kit (Bio-Rad, Hercules, CA, USA). Denatured proteins were separated by sodium dodecyl sulfate-polyacrylamide gel electrophoresis (SDS-PAGE) on 12% acrylamide gels, and then transferred to Hybond-membranes (Amersham, Germany). The membranes were blocked overnight in 5% skimmed milk in Tris-buffered saline with Tween 20 (TBST; 10 mM Tris-HCl, 150 mM NaCl, 0.1% Tween 20). For immunoblotting, the membranes were incubated for 1 h with the primary antibody, rinsed with TBST and incubated with anti-rabbit, anti-mouse or anti-goat IgG antibodies conjugated to horseradish peroxidase (HRP; Dako, Carpinteria CA, USA) at a dilution of 1∶1000. After applying enhanced chemiluminescent (ECL)-Plus detection reagents (Santa Cruz Biotechnology, Santa Cruz, CA, USA), the protein bands were visualized using an X-ray film (Fujifilm, Tokyo, Japan). The immunoblots were washed with western blotting (WB) stripping buffer (pH 2–3; Nacalai, Tokyo, Japan) and probed using a monoclonal antibody specific for β-actin (1∶1000; Santa Cruz Biotechnology, Santa Cruz, CA, USA).

### Immunofluorescence

Cells were grown on glass coverslips, fixed with PBS containing 4% formaldehyde for 10 min and permeabilized with 0.2% Triton X-100 in PBS for 10 min at room temperature. After washing with PBS, the cells were incubated overnight at 4°C with Alexa Fluor 594 Phalloidin (Invitrogen, Carlsbad, CA, USA) to enable the lamellipodia to be visualized. Nuclei were stained with 1 µg mL^−1^ (DAPI; Sigma–Aldrich St Louis, MO, USA) for 15 min at 37°C. The coverslips were then mounted with SlowFade Gold Antifade Reagent (Invitrogen, Carlsbad, CA, USA) and observed under a confocal laser microscope (Olympus, Tokyo, Japan).

### Statistical analysis

Statistical evaluation was performed using Spearman's rank correlation coefficient to analyze ranked data, and the Mann–Whitney U test to differentiate the means of different groups. A *p*-value of <0.05 was considered statistically significant. SPSS v. 10.0 software (SPSS, Chicago, IL, USA) was employed to analyze all data.

## Results

### The effects of anacardic acids on the ovarian carcinoma cells

**Figure 1:**
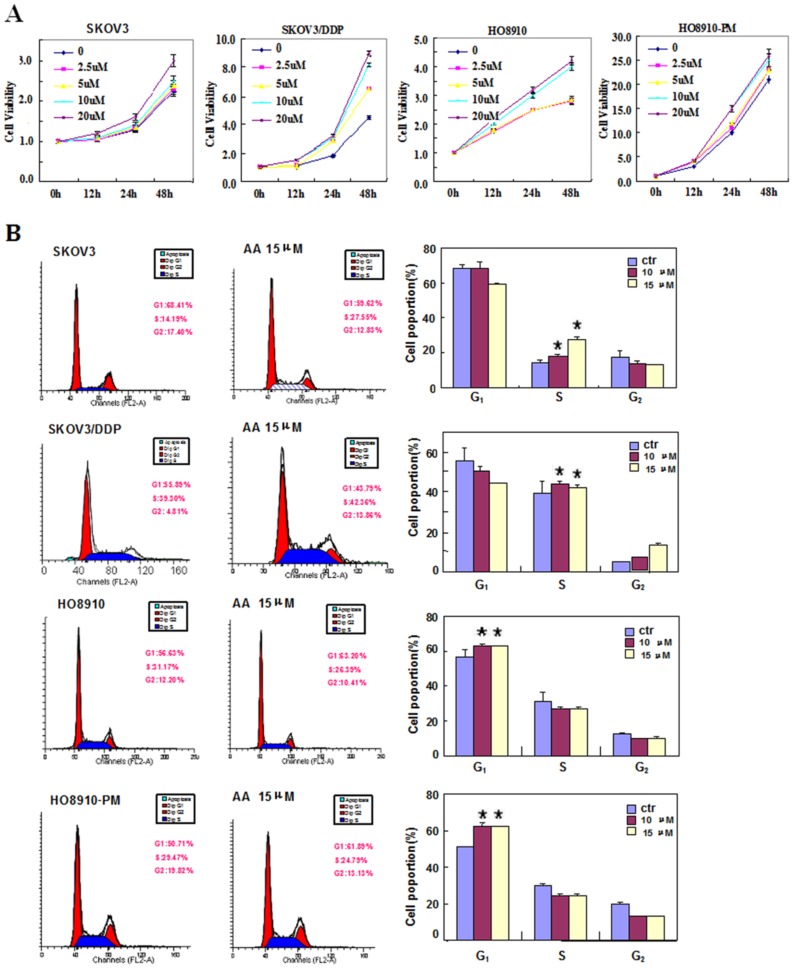
The effects of anacardic acids on the proliferation and cell cycle of ovarian carcinoma cells. CCK-8 cell proliferation assays show that AA treatment of SKOV3 and SKOV3/DDP, and HO8910 and HO8910-PM cell-line pairs induces cell proliferation (A). AA treatment induced S proliferation in SKOV3 and SKOV3/DDP cells, and G1 arrest in HO8910 and HO8910-PM cells (B). * *p<0.05*. Results are representative of three separate experiments; data are expressed as the mean ± standard deviation.

**Figure 2:**
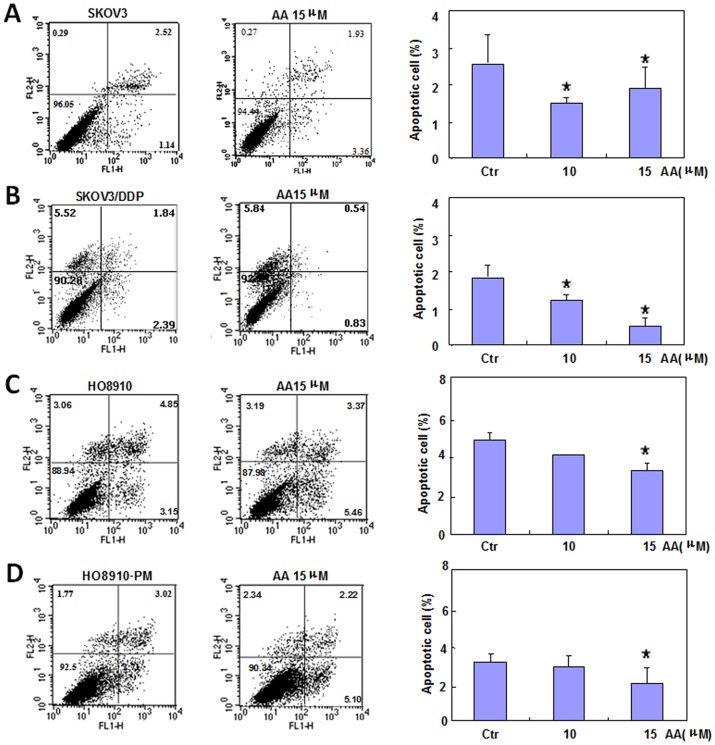
The effects of anacardic acids on the apoptosis of ovarian carcinoma cells. Flow cytometric apoptosis analysis shows that AA treatment inhibits late apoptosis in SKOV3, SKOV3/DDP, HO8910 and HO8910-PM cells. * *p<0.05*. Results are representative of three separate experiments; data are expressed as the mean ± standard deviation.

**Figure 3:**
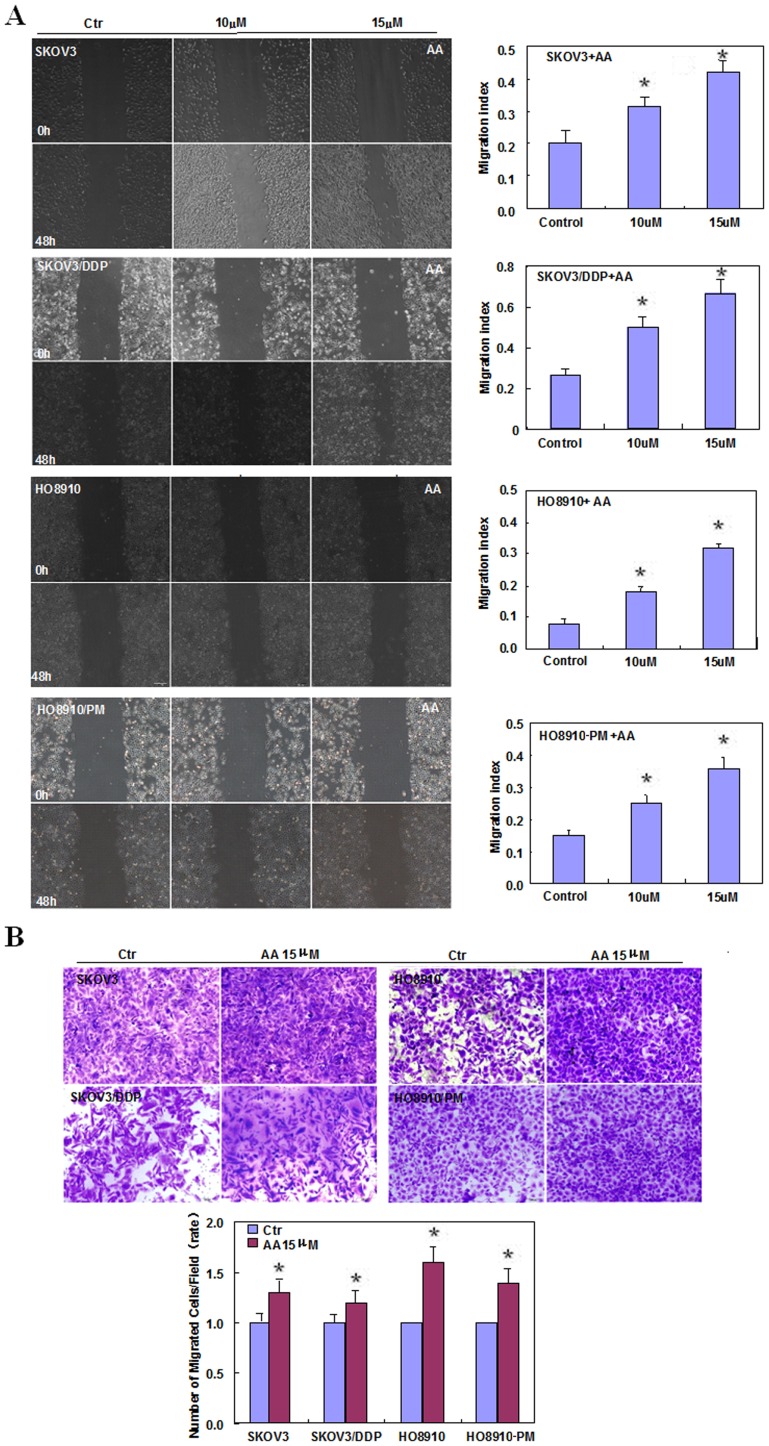
Effects of anacardic acids on the invasive and metastatic ability of ovarian carcinoma cells. Wound-healing assays show that AA treatment increases the ability of SKOV3, SKOV3/DDP, HO8910 and HO8910-PM cells to migrate in a concentration-dependent manner (**A**). Treated cells also exhibit invasive potential (**B**). * *p<0.05*. Results are representative of three separate experiments; data are expressed as the mean ± standard deviation.

**Figure 4:**
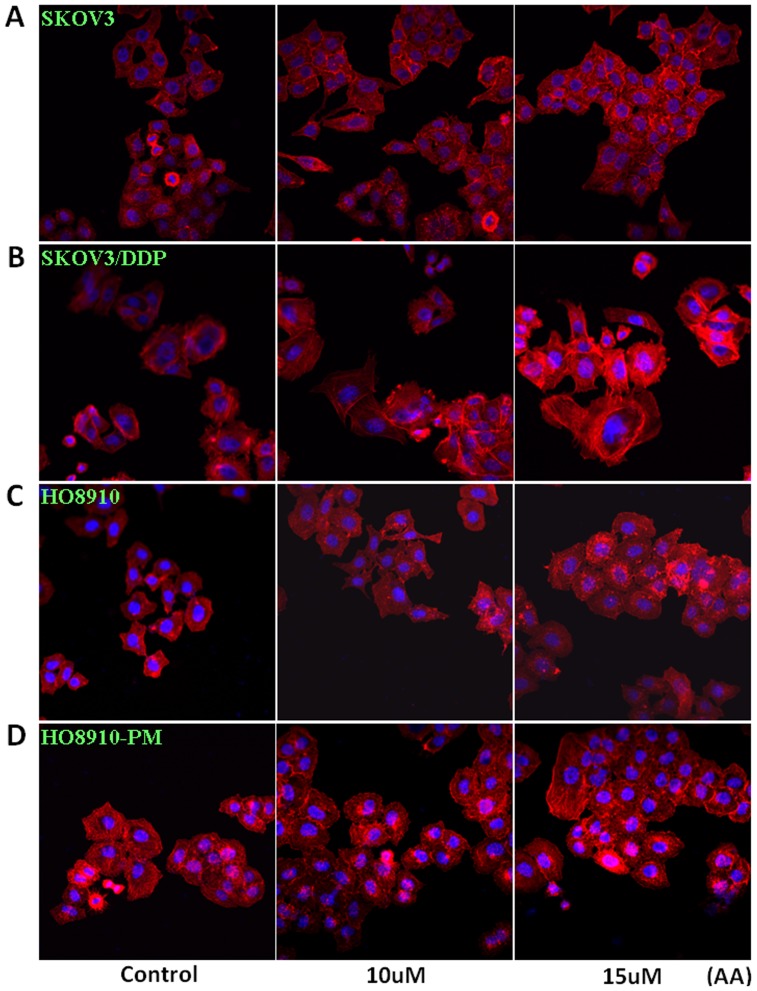
Effects of anacardic acids on the lamellipodia formation of ovarian carcinoma cells. AA exposure induces lamellipodia formation in all four cell lines as indicated by the F-actin structure.

Two pairs of cell lines, SKOV3 and SKOV3/DDP cells, and HO8910 and HO8910-PM cells, were exposed to AA (0 µM, 2.5 µM, 5 µM, 10 µM, 15 µM, 20 µM)and subjected to CCK-8 proliferation assays (Figure 1A, *p<0.05*). SKOV3, SKOV3/DDP, HO8910 and HO8910-PM cells showed stronger proliferation when treated with AA compared with control cell lines. We found that the function of AA in promoting proliferation was positive correlated with its concentration, 10 µM AA can promote proliferation significantly, then we select 10 µM, 15 µM, 20 µM as experimental conditions to complete the following experiment. AA treatment induced S proliferation in SKOV3 and SKOV3/DDP cells; conversely, AA induced G1 arrest in HO8910 and HO8910-PM cells (Figure 1B, *p<0.05*). Flow cytometric apoptosis analysis showed that AA treatment (15 µM) inhibited late apoptosis in SKOV3, SKOV3/DDP, HO8910 and HO8910-PM cells (Figure 2, *p<0.05*), and wound-healing and invasion assays showed that AA promoted cell migration and invasion in a concentration-dependent manner (Figure 3A and B, *p<0.05*). In addition, AA exposure induced lamellipodia formation in all four cell lines, as indicated by the F-actin structure (Figure 4).

### The mRNA and protein expression of phenotype-related molecules in ovarian carcinoma cells after exposure to AA

**Figure 5:**
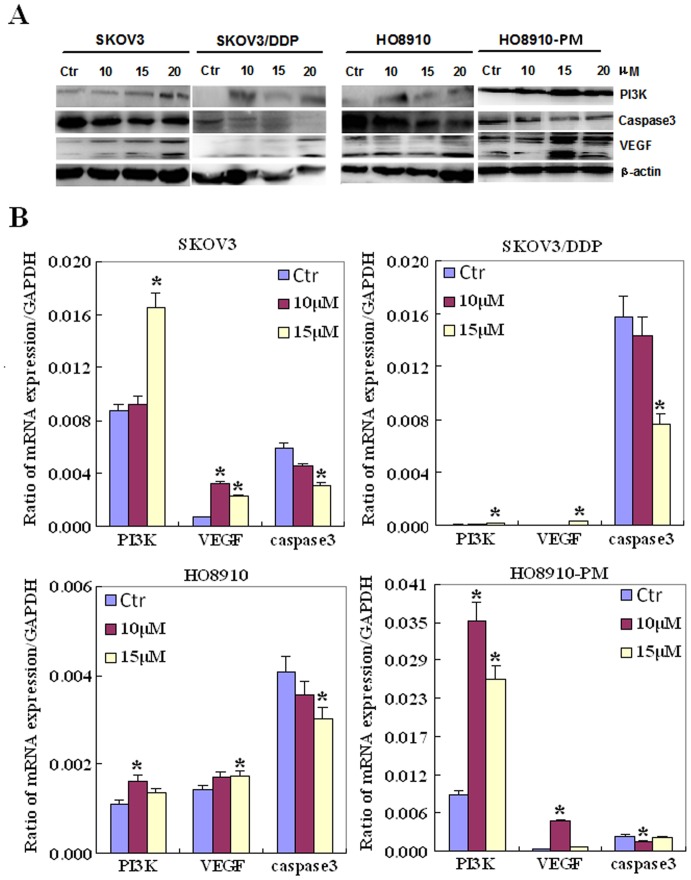
The mRNA and protein expression of ovarian carcinoma cells treated with anacardic acids. Effects of AA exposure on the mRNA expression levels of proliferation and apoptosis-related molecules, and their corresponding proteins, in the ovarian carcinoma cell-line pairs using real-time RT-PCR (**A**) and western blot analysis (**B**). * *p*<0.05. Results are representative of three separate experiments; data are expressed as the mean ± standard deviation.

After treatment with AA, the mRNA expression levels of caspase 3 in SKOV3, SKOV3/DDP, HO8910 and HO8910-PM cell lines were lower than those observed in control cells. In contrast, the mRNA expression levels of PI3K and VEGF were higher than those of control cells (Figure 5A, *p<0.05*). Western blot analysis of the protein expression levels showed that AA exposure up-regulated PI3K and VEGF protein expression, and down-regulated caspase 3 protein expression in both cell-line pairs (Figure 5B, *p<0.05*).

## Discussion

AA is commonly found in plants of the Anacardiaceae family and is a dietary component found in cashew apple (*Anacardium occidentale*) and ginkgo (*Ginkgo biloba*) leaves and fruits and is found in a number of medicinal plants that have potential activity against cancer cell lines [14]–[15]. Several studies have reported that AA plays an anticancer role, which may associated with the reduced expression of survivin and X-linked inhibitor of apoptosis protein, which are anti-apoptotic proteins associated with cellular survival and radioresistance, and the radiosensitization of pituitary adenoma cells [16]. AA also plays an antibacterial role and a previous study focused on structure–activity relationships revealed that the carboxyl group on the aromatic ring and an unsaturated side chain in the anacardic acid derivative are important for motility inhibition and the lytic activity of AA against zoospores [17]. It also plays a role as an anti-oxidant and demonstrates anti-obesity and anti-inflammatory activities [18]–[20].

AA inhibits the activation of both the inducible and constitutive expression of nuclear factor-kappa B (NF-kB), which is activated by carcinogens and growth factors, and is thought to potentiate apoptosis in tumor cells at a concentration of 25 µM [9]. Meanwhile, a previous study has shown via a series of investigations, such as the detection of cytotoxicity, the expression of relevant proteins and Ca^2+^ mobility, that AA induces ER(Endoplasmic Reticulum) stress and autophagy in lung cancer cells at a concentration of 10 µM [10]. Wound healing migration assays, Transwell migration assays and mouse model assays have additionally suggested that AA may function as a potent tumor angiogenesis inhibitor by targeting the Src/FAK/Rho GTPase signaling pathway, leading to significant suppression of prostate tumor growth at low dosage levels (5–50 µM) [11]. AA has been shown by a series of experiments, including cell cycle analysis, RT-PCR and WB, to inhibit LNCaP cell proliferation by inducing G1/S cell cycle arrest and apoptosis at concentrations of 5, 25, 125 µM [12]. However, AA has not been applied in clinical situations, although many people are thought to take it as a healthcare supplement. It should be noted that, in contrast to some other cancers, our present study suggests that AA may promote proliferation and inhibit apoptosis in ovarian cancer cells, indicating that the function of AA in ovarian cancer cell lines is a controversial issue.

The related molecular mechanisms of AA in ovarian carcinoma cells has, to date, remained unknown. To investigate the related mechanisms in ovarian cancer cells, we selected two pairs of ovarian carcinoma cell lines based on their drug resistance and invasive abilities (SKOV3 and SKOV3/DDP, and HO8910 and HO8910-PM, respectively). Our experimental studies showed that AA significantly induced cell viability in ovarian cancer cells at 15 µM. It should be noted that, although AA induced cell proliferation in all cells in a concentration- and time-dependent manner, both the drug-resistant (SKOV3/DDP) and highly invasive (HO8910-PM) cells showed greater sensitivity to AA exposure, which may be due to their greater proportion of stem cells (tumor-initiating cells) or the increased stem cell-like properties of these cells. AA treatment induced S proliferation in SKOV3 and SKOV3/DDP cells; conversely, it induced G1 arrest in HO8910 and HO8910-PM cells. Flow cytometric apoptosis analysis showed that treatment with AA inhibited late apoptosis in SKOV3, SKOV3/DDP, HO8910 and HO8910-PM cells. There was a case reported, in breast cancer cell, AA may preferentially inhibit ER-α (estrogen receptor-α)-positive breast cancer cell proliferation by direct estrogen receptor DNA binding domain (ER DBD) interaction[13]. We further complete immunofluoresce experiment to detect the expression of ER in four ovarian cancer cell lines, the result showed that four ovarian cancer cell lines all have ER expression. Then we suggest that ER-negative or ER-positive may not effect AA's function on ovarian cancer cell lines, the difference between AA's function on breast cancer cell lines and ovarian cancer cell lines may through different routes. To investigate this finding further, we assessed apoptosis pathway-related indicator, caspase 3 [25]–[27], and found that its mRNA and protein expression levels were significantly lower in cells exposed to AA. In disagreement with some experiments [28]–[31]. Consequently, we considered that AA may have a pro-tumor function in ovarian carcinoma.

To study this finding further, we examined a number of other indicators and found that the expressions of PI3K mRNA and protein in ovarian cancer cells treated with AA were higher when compared to untreated control cells. Our data are in accordance with that of Zachary et al. [21], who reported that the PI3K/AKT/mTOR pathway is diverse and affects equally diverse aspects of ovarian tumor development, progression and patient survival. Several experiments have been conducted to investigate targeted cancer therapy via the PI3K pathway. Indeed, a recent prospective, large-scale genomic analysis has shown that the PI3K/AKT pathway is frequently deregulated in high-grade serous ovarian tumors [22]–[23]. PI3K can be considered as a major mediator of survival signals that protect ovarian cancer cells from apoptosis induction [24]. For this reason, we presume that AA may promote proliferation in ovarian cancer via the PI3K pathway. Furthermore, our study has shown that AA promotes cell migration and invasion in a concentration-dependent manner, and AA exposure induces lamellipodia formation in all four cell lines studied, as indicated by the closely concentrated F-actin structure.

We also detected invasion and metastasis-related indicators and found that the expression levels of VEGF mRNA and protein in AA-treated cells increased compared to control cells. VEGF is well known as one of the main growth factors involved in vessel formation [32], [33]. Evidence suggests that the abnormal expression of VEGF in ovarian cancer is closely associated with tumor invasion and metastasis [34], [35]. As we have discussed above, AA plays a diverse role in various cancers and acts through multiple pathways. For example, the potent inhibitory activity of AA on VEGF-stimulated migration, capillary structure formation, attachment and paxillin activation in endothelial cells has previously been reported [11]. However, in our study, AA was found to promote the expression of VEGF mRNA and protein in ovarian cancer cells. Therefore, we speculate that AA may promote migration and invasion in ovarian cancer via the VEGF pathway.

In conclusion, we suggest that AA may potentiate proliferation, invasion and metastasis via the PI3K and VEGF pathways in ovarian cancer cell lines. However, the aberrant specific molecular mechanisms of AA in ovarian carcinoma need further study and its clinical manipulation also needs to be cautiously considered in future work.

## Supporting Information

Table S1 **Primer sequences selected for real-time RT-PCR**
(DOC)

## References

[1] MantiaSmaldone GM, Edwards RP, Vlad AM (2011) Targeted treatment of recurrent platinum-resistant ovarian cancer: current and emerging therapies. Cancer Manag Res. doi: 10.2147/CMR.S8759. 10.2147/CMR.S8759 PMC3130354 21734812

[2] Lengyel E (2010) Ovarian cancer development and metastasis. Am J Pathol. doi: 10.2353/ajpath.2010.100105. 10.2353/ajpath.2010.100105 PMC2928939 20651229

[3] Eisenkop SM, Friedman RL, Wang HJ (1998) Complete cytore-ductive surgery is feasible and maximizes survival in patients with advanced epithelial ovarian cancer: a prospective study. Gynecol Oncol 69: 103–108. 9600815 10.1006/gyno.1998.4955

[4] Ji K, Ye L, Mason MD, Jiang WG (2013) The Kiss1/Kiss1R complex as a negative regulator of cell motility and cancer metastasis (Review). Int J Mol Med. Oct 32(4): 747–54. 10.3892/ijmm.2013.1472 23969598

[5] Sowmyalakshmi S, Nur M, Akbarsha MA, Thirugnanam S, Rohr J, et al (2005) Investigation on Semecarpus Lehyam—a Siddha medicine for breast cancer. Planta 220: 910–8. 15517350 10.1007/s00425-004-1405-4

[6] Rea AI, Schmidt JM, Setzer WN, Sibanda S, Taylor C, et al (2003) Cytotoxic activity of Ozoroa insignis from Zimbabwe. Fitoterapia 74: 732–5. 14630185 10.1016/j.fitote.2003.08.007

[7] Toyomizu M, Okamoto K, Ishibashi T, Chen Z, Nakatsu T (2000) Uncoupling effect of anacardic acids from cashew nut shell oil on oxidative phosphorylation of rat liver mitochondria. Life Sciences, 66: , 229–234. 10.1016/s0024-3205(99)00585-8 10665998

[8] Sun Y, Jiang X, Chen S, Price BD (2006) Inhibition of histone acetyltransferase activity by anacardic acid sensitizes tumor cells to ionizing radiation. FEBS Letters, 580: , 4353–4356. 10.1016/j.febslet.2006.06.092 16844118

[9] Sung B, Pandey MK, Ahn KS, Yi T, Chaturvedi MM, et al. (2008) Anacardic acid (6-nonadecyl salicylic acid), an inhibitor of histone acetyltransferase, suppresses expression of nuclear factor-kappaB-regulated gene products involved in cell survival, proliferation, invasion, and inflammation through inhibition of the inhibitory subunit of nuclear factor-kappaB alpha kinase, leading to potentiation of apoptosis. Blood, 111: , 4880–4891. 10.1182/blood-2007-10-117994 PMC2384122 18349320

[10] Seong YA, Shin PG, Yoon JS, Yadunandam AK, Kim GD (2013) Induction of the Endoplasmic Reticulum Stress and Autophagy in Human Lung Carcinoma A549 Cells by Anacardic Acid. Cell Biochem Biophys 013-9717-2. 10.1007/s12013-013-9717-2 23955513

[11] Wu Y, He L, Zhang L, Chen J, Yi Z, et al (2011) Anacardic Acid (6-Pentadecylsalicylic Acid) Inhibits Tumor Angiogenesis by Targeting Src/FAK/Rho GTPases Signaling Pathway. JPET 339: 403–411. 10.1124/jpet.111.181891 21828260

[12] Tan J, Chen B, He L, Tang Y, Jiang Z, et al (2012) Anacardic acid (6-pentadecylsalicylic acid) induces apoptosis of prostate cancer cells through inhibition of androgen receptor and activation of p53 signaling. Chin J Cancer Res 24(4): 275–283. 23359208 10.3978/j.issn.1000-9604.2012.10.07 PMC3551327

[13] Schultz DJ, Wickramasinghe NS, Ivanova MM, Isaacs SM, Dougherty SM, et al (2010) Anacardic acid inhibits estrogen receptor alpha-DNA binding and reduces target gene transcription and breast cancer cell proliferation. Mol Cancer Ther. March 9(3): 594–605. 10.1158/1535-7163.MCT-09-0978 PMC2837512 20197399

[14] Chattopadhyaya MK, Khare RL (1969) Isolation of anacardic acid from *Semicarpus Anacardium* Linn. And study of its anthelmintic activity. Indian J Pharm 31: 104–105p.

[15] Cassady JM, Chang CJ, JL M (1981) Recent advances in the isolation and structural elucidation of antineoplastic agents of higher plants. Natural products as medicinal agents: Plenary lectures of the International Research Congress on Medicinal Plant Research. Hippokrates Verlag: Stuttgart; 93–124p.

[16] SukumariRamesh S, Singh N, Jensen MA, Dhandapani KM, Vender JR (2011) Anacardic acid induces caspase independent apoptosis and radiosensitizes pituitary adenoma cells laboratory investigation. Journal of Neurosurgery, 114: , 1681–1690. 10.3171/2010.12.JNS10588 21275565

[17] Begum P, Hashidoko Y, Islam MT, Ogawa Y, Tahara S (2002) Zoosporicidal activities of anacardic acids against Aphanomyces cochlioides. Z Naturforsch C. 57: , 874–882. 10.1515/znc-2002-9-1020 12440727

[18] Trevisan MT, Pfundstein B, Haubner R, Wurtele G, Spiegelhalder B, et al. (2006) Characterization of alkyl phenols in cashew (Anacardium occidentale) products and assay of their antioxidant capacity. Food and Chemical Toxicology, 44: , 188–197. 10.1016/j.fct.2005.06.012 16095792

[19] Toyomizo M, Okamato K, Ishibashi T, Nakatsu T, Akiba Y (2003) Reducing effect of dietary anacardic acid on body fat pads in rats. Anim Sci J, 74: , 499–504.

[20] Shobha SV, Ramadoss CS, Ravindranath B (1994) Inhibition of soybean lipoxygenase -1 by anacardic acids, cardols, and cardanols. Journal of Natural Products, 57: , 1755–1757.

[21] Dobbin ZC, Landen CN (2013) The Importance of the PI3K/AKT/MTOR Pathway in the Progression of Ovarian Cancer. Int. J. Mol. Sci. *14*: , 8213–8227. 10.3390/ijms14048213 PMC3645739 23591839

[22] Cejka D, Kuntner C, Preusser M, FritzerSzekeres M, Fueger BJ, et al. (2009) FDG uptake is a surrogate marker for defining the optimal biological dose of the mTOR inhibitor everolimus in vivo. Br J Cancer 100: , 1739–1745. 10.1038/sj.bjc.6605076 PMC2695687 19436299

[23] Altomare DA, Wang HQ, Skele KL, De Rienzo A, KleinSzanto AJ, et al. (2004) AKT and mTOR phosphorylation is frequently detected in ovarian cancer and can be targeted to disrupt ovarian tumor cell growth. Oncogene 23: , 5853–5857. 10.1038/sj.onc.1207721 15208673

[24] Mazzoletti M, Broggini M (2010) PI3K/AKT/mTOR inhibitors in ovarian cancer. Curr Med Chem 17: , 4433–4447. 10.2174/092986710794182999 21062259

[25] Lin MY, Lee YR, Chiang SY, Li YZ, Chen YS, et al (2013) Cortex Moutan Induces Bladder Cancer Cell Death via Apoptosis and Retards Tumor Growth in Mouse Bladders. Evid Based Complement Alternat Med 2013: 207279. 24282433 10.1155/2013/207279 PMC3824643

[26] Gurunathan S, Raman J, Malek SN, John PA, Vikineswary S (2013) Green synthesis of silver nanoparticles using Ganoderma neo-japonicum Imazeki: a potential cytotoxic agent against breast cancer cells. Int J Nanomedicine 8: 4399–413. 24265551 10.2147/IJN.S51881 PMC3833323

[27] Yu B, Yue DM, Shu LH, Li NJ, Wang JH (2013) Pseudolaric acid B induces caspase-dependent cell death in human ovarian cancer cells. Oncol Rep. Nov 25. doi: 10.3892/or.2013.2869. 10.3892/or.2013.2869 24276652

[28] Sung B, Pandey MK, Ahn KS, Yi T, Chaturvedi MM, et al.. (2008) Anacardic acid (6-nonadecyl salicylic acid), an inhibitor of histone acetyltransferase, suppresses expression of nuclear factor-κB–regulated gene products involved in cell survival, proliferation, invasion, and inflammation through inhibition of the inhibitory subunit of nuclear factor-κBα kinase, leading to potentiation of apoptosis. Blood May 15, vol. 111 no. 10 4880–4891. 10.1182/blood-2007-10-117994 PMC2384122 18349320

[29] Seong YA, Shin PG, Kim GD (2013) Anacardic acid induces mitochondrial-mediated apoptosis in the A549 human lung adenocarcinoma cells. Int J Oncol Mar 42(3): 1045–51. 10.3892/ijo.2013.1763 23314312

[30] KusioKobialka M, DudkaRuszkowska W, Ghizzoni M, Dekker FJ, Piwocka K (2013) Inhibition of PCAF by anacardic acid derivative leads to apoptosis and breaks resistance to DNA damage in BCR-ABL-expressing cells. Anticancer Agents Med Chem. Jun 13(5): 762–7. 10.2174/1871520611313050010 23157591

[31] SukumariRamesh S, Singh N, Jensen MA, Dhandapani KM, Vender JR (2011) Anacardic acid induces caspase-independent apoptosis and radiosensitizes pituitary adenoma cells J Neurosurg. Jun 114(6): 1681–90. 10.3171/2010.12.JNS10588 21275565

[32] Kajdaniuk D, Marek B, BorgielMarek H, Kos-Kud B (2011) Vascular endothelial growth factor (VEGF) - part 1: in physiology and pathophysiology. Endokrynol Pol. 62: 444–455. 22069106

[33] Yang Zhao, HuaChuan Zheng, Shuo Chen, Wen-Feng Gou, Li-Jun Xiao, et al (2013) The role of RhoC in ovarian epithelial carcinoma: A marker for carcinogenesis, progression, prognosis, and target therapy. Gynecologic Oncology 130: 570–578. 23764197 10.1016/j.ygyno.2013.06.004

[34] DinizBizzo SM, Meira DD, Lima JM, Mororó Jda S, CasalidaRocha JC, et al (2010) Peritoneal VEGF burden as a predictor of cytoreductive surgery outcome in women with epithelial ovarian cancer. Int J Gynaecol Obstet 109: 113–117. 20167321 10.1016/j.ijgo.2009.11.021

[35] Hefler LA, Zeillinger R, Grimm C, Sood AK, Cheng WF, et al (2006) Preoperative serum vascular endothelial growth factor as a prognostic parameter in ovarian cancer. Gynecol Oncol 103: 512–517. 16750560 10.1016/j.ygyno.2006.03.058

