# Supplementary material for: Anacardic Acid Enhances the Proliferation of Human Ovarian Cancer Cells
Source: PLoS One. 2014 Jun 12;9(6):e99361. doi: 10.1371/journal.pone.0099361 (PMC4055655; doi:10.1371/journal.pone.0099361)
Supplement: Table S1 — Primer sequences selected for real-time RT-PCR (DOC) [file pone.0099361.s001.doc]

**Table S1.** Primer sequences selected for real-time RT-PCR

| **Name** | **Primer sequence** | **Distribution** | **AT**  **(^o^C)** | **Product**  **size (bp)** | **Extension**  **time (s)** |
| --- | --- | --- | --- | --- | --- |
| *Caspase-3* | *F: 5'- TGGCATTGAGACAGACA-3'*  *R: 5'- GGCACAAAGCGACTG-3'* | *NM_004346*  *773-926* | *60* | *154* | *34* |
| *VEGF* | *F: 5'- AGGAGGAGGGCAGAATC-3'*  *R: 5'- ATGTGCTGGCCTTGGT-3'* | *NM_001171630*  *1131-1388* | *60* | *258* | *34* |
| *PI3K* | *F: 5' - CACCGCATTTGTCGT -3'*  *R:5' - CTCCCACTTCTACGC -3'* | *NM_001242466*  *4016-4240* | *60* | *225* | *34* |
| *GAPDH* | *F: 5'- CAATGACCCCTTCATTGACC-3'*  *R: 5'- TGGAAGATGGTGATGGGATT-3'* | *NM_ 002046.3*  *201-335* | *60* | *135* | *34* |

AT = annealing temperature
